# Efavirenz Promotes β-Secretase Expression and Increased Aβ_1-40,42_ via Oxidative Stress and Reduced Microglial Phagocytosis: Implications for HIV Associated Neurocognitive Disorders (HAND)

**DOI:** 10.1371/journal.pone.0095500

**Published:** 2014-04-23

**Authors:** Lecia A. M. Brown, Jingji Jin, Darren Ferrell, Edin Sadic, Demian Obregon, Adam J. Smith, Jun Tan, Brian Giunta

**Affiliations:** 1 Department of Psychiatry and Behavioral Neurosciences, Neuroimmunology Laboratory, University of South Florida, Morsani College of Medicine, Tampa, Florida, United States of America; 2 Department of Molecular Medicine, University of South Florida, Morsani College of Medicine, Tampa, Florida, United States of America; 3 Department of Psychiatry and Neurosciences, Rashid Developmental Neurobiology Laboratory, Silver Child Development Center, University of South Florida, Morsani College of Medicine, Tampa, Florida, United States of America; 4 Center of Excellence for Aging and Brain Repair, Department of Neurosurgery and Brain Repair, University of South Florida, Morsani College of Medicine, Tampa, Florida, United States of America; 5 James A. Haley Veterans Administration Hospital, Tampa, Florida, United States of America; Virginia Commonwealth University, United States of America

## Abstract

Efavirenz (EFV) is among the most commonly used antiretroviral drugs globally, causes neurological symptoms that interfere with adherence and reduce tolerability, and may have central nervous system (CNS) effects that contribute in part to HIV associated neurocognitive disorders (HAND) in patients on combination antiretroviral therapy (cART). Thus we evaluated a commonly used EFV containing regimen: EFV/zidovudine (AZT)/lamivudine (3TC) in murine N2a cells transfected with the human “Swedish” mutant form of amyloid precursor protein (SweAPP N2a cells) to assess for promotion of amyloid-beta (Aβ) production. Treatment with EFV or the EFV containing regimen generated significantly increased soluble amyloid beta (Aβ), and promoted increased β-secretase-1 (BACE-1) expression while 3TC, AZT, or, vehicle control did not significantly alter these endpoints. Further, EFV or the EFV containing regimen promoted significantly more mitochondrial stress in SweAPP N2a cells as compared to 3TC, AZT, or vehicle control. We next tested the EFV containing regimen in Aβ - producing Tg2576 mice combined or singly using clinically relevant doses. EFV or the EFV containing regimen promoted significantly more BACE-1 expression and soluble Aβ generation while 3TC, AZT, or vehicle control did not. Finally, microglial Aβ phagocytosis was significantly reduced by EFV or the EFV containing regimen but not by AZT, 3TC, or vehicle control alone. These data suggest the majority of Aβ promoting effects of this cART regimen are dependent upon EFV as it promotes both increased production, and decreased clearance of Aβ peptide.

## Introduction

There has been considerable growth in patient's receiving combination antiretroviral therapy (cART) in recent years [1]. Up to 50% of long-term HIV-infected patients experience HIV associated neurocognitive disorders (HAND) [2]. Most recently it was shown the Non-Nucleoside Reverse Transcriptase Inhibitor (NNRTI) efavirenz (EFV) is associated with cognitive disorders even in asymptomatic HIV-infected patients [3]. A randomized controlled study [4] found subjects receiving EFV-containing regimens showed less improvement from baseline on instruments examining speed of information processing and executive function than patients not on EFV. Further, patients with preserved immune function on EFV regimens showed greater improvement on Trail-Making Tests A and B and the Wechsler Adult Intelligence Scale digital symbol test after EFV interruption than the non-EFV control group [5]. EFV has substantial rates of central nervous system (CNS) side effects aside of cognitive impairment including sleep and dreaming disturbances and anxiety [6]–[8] that interfere with adherence and tolerability as well [9].

Amyloid-beta (Aβ) peptide generation and aggregation as plaques are traditionally known as key events in the development of Alzheimer's Disease (AD; [10]–[13]). The peptides have been evidenced to be neurotoxic, as they are reported mediators of inflammation [14], [15], and oxidative stress [16]. Aβ peptides are produced *via* the amyloidogenic pathway of amyloid precursor protein (APP) proteolysis, which involves the actions of β and γ-secretases [13], [17]. Initially, β-secretase (BACE-1) cleaves APP, creating an Aβ-containing carboxyl-terminal fragment known as β-C-terminal fragment (β-CTF) [18]. This proteolysis also generates an amino-terminal, soluble APP-β (sAPP-β) fragment, which is released extracellularly. Intracellularly, β-CTF is then cleaved by a multi-protein γ-secretase complex that results in generation of the Aβ peptide and a smaller γ-CTF [19], [20]. In the human brain Aβ_1-40_ is the predominant form whereas Aβ_1-42_ represents about 10% of Aβ in brain and has a greater propensity to form neurotoxic oligomeric and aggregated species [21].

The rapid, early clinical phase-in of cART required dose de-escalations secondary to toxicities suggested to be related to mitochondrial drug side effects [22]. Mitochondrial dysfunction can result in an elevation of reactive oxygen species (ROS) that in turn promotes amyloidogenic APP processing by promoting BACE-1 activity [18]. Such mitochondrial stress has also been reported occurs in patients taking lamivudine (3TC), zidovudine (AZT) and especially EFV [23]–[28]; a commonly used cART regimen [29]–[31]. In light of the increasing life-span's imparted by cART, the mitochondrial promoted by cART [23]–[27], [32]–[34], and the age associated risk for developing amyloid pathology [35], it is not surprising that a body of epidemiological data suggests significant numbers of long-term HIV survivors are at elevated risk of developing early brain aging in the form of AD like pathology including Aβ deposition [36]–[42].

As a result, we hypothesized that Aβ pathology may be produced *via* the amyloidogenic pathway of APP proteolysis, which involves the actions of BACE-1 [13] in patients on such regimen and sought to test this with *in vitro* and *in vivo* models. Our results indicate that EFV is the primary antiretroviral in this commonly used EFV containing regimen: EFV/3TC/AZT [29]–[31] which is responsible for its promotion of Aβ pathology.

## Materials and Methods

All animal work was approved by the University of South Florida Institutional Animal Care and Use Committee (IACUC).

### Reagents

Aβ_1-40_ and Aβ_1-42_ peptides and control peptide (Aβ_40-1_) were obtained from QCB (Hopkinton, MA) and freshly solubilized in distilled H_2_O immediately before use. To determine the oligomeric state of Aβ in our assays, Aβ was immunoprecipitated from cell supernatants after incubation with microglia and/or neurons, and Western blot analysis was performed at time points of 12, 24, and 48 hr. Data revealed that both Aβ_1-40_ and Aβ_1-42_, irrespective of the time points assayed, existed as a ladder of SDS-stable oligomers, with a predominant species of ∼32 kDa. Immun-Blot polyvinylidene difluoride (PVDF) membranes were purchased from Bio-Rad (Hercules, CA). Tris-buffered saline was obtained from Bio-Rad (Hercules, CA) and luminol reagent was obtained from Pierce Biotechnology. Anti-actin antibody was obtained from Roche. Antiretrovirals were obtained from The National Institutes of Health (NIH) AIDS Research and Reference Reagent Program (Rockville, MD). Regarding dosages administered, cART effects *in vivo* are likely to occur over long- term exposures [43]. Thus, chronic, low dose, *in vivo* effects of any reagent are often very appropriately modeled *in vitro*, by proportionally higher doses of the same reagent, over more acute time frames [43]. For these reasons we used 10 µM cART doses throughout our *in vitro* works and per our previous study [44]. The doses of cART administered *in vivo* were based on based on human clinical therapy [45], the body weight of the mice, the short dosing period of 10 ten days, the administration method being in chow as opposed to intravenous administration, as well as those reported in previous publications: AZT 50 mg/kg [46]-[48], 3TC 40 mg/kg [47], [48], and EFV 15 mg/kg [46], [49].

### Neuronal Aβ Production Assay

This was performed according to our previous works [44]. Briefly, SweAPP N2a cells were treated with EFV, AZT, and 3TC both alone (10 µM) and in combination (10 µM) for 18 hours. Aβ_1-40, 42_ peptides were detected directly from the conditioned media and quantified in these samples using Aβ_1-40, 42_ ELISA kits (Life Technologies) in accordance with the manufacturer's instructions.

### Western immunoblotting

Western blot was performed as described previously [50], [51]. Briefly, total protein content was estimated using the Bio-Rad protein assay in strict accordance with manufacturer's directions. Immunoblotting was performed with a primary antibody followed by an anti-mouse HRP-conjugated IgG secondary antibody as a tracer. Primary antibodies used included: 6E10 monoclonal anti-Aβ antibody (Covance, 1∶1000), polyclonal Rabbit anti- BACE-1 (Sigma1∶1000), C-terminus monoclonal anti-BACE-1 (Millipore 1∶1000), and anti-actin antibody (Sigma, 1∶1500).

For the *in vivo* studies of Aβ associated pathology we employed our previous methods [50], [51]. Left hemispheres of 3 month old transgenic and nontransgenic mouse brains were lysed in ice-cold lysis buffer and aliquots were electrophoretically separated using 16.5% Tris–tricine gels. Electrophoresed proteins were then transferred to PVDF membranes (Bio-Rad), washed in dH_2_O, then blocked in Tris-buffered saline containing 5% (w/v) non-fat dry milk. Membranes were then hybridized with various primary antibodies followed by washing in dH_2_O and then incubation for 1 h at ambient temperature with the appropriate HRP-conjugated secondary antibody (1∶1000). For both *in vitro* and *in vivo* studies, blots were developed and then assessed densitometrically analyzed using the Fluor-S MultiImager with Quantity One software (Bio-Rad).

### Mitochondrial Stress Analysis: Adenosine triphosphate (ATP), mitochondrial membrane potential (MMP), and reactive oxygen species (ROS)

ATP determination was performed using the Invitrogen ATP determination kit (A22066). MMP analysis was performed using a JC-1 (excitation filter 530/25, emission filter 590/35) MMP detection kit (Biotium). Cellular ROS generation was analyzed using 2,7-dichloro dihydrofluorescein diacetate (excitation filter 485/20, emission filter528/20) from the Invitrogen ROS detection kit. For all three analyses of mitochondrial stress, the reagents and reaction mixture were combined according to the supplied protocol. All fluorescence measurements were read using a Biotek Synergy H1 microplate reader.

### Microglial Phagocytosis Assay

This was performed according to our previous studies [44], [50]. Briefly, primary mouse microglia were treated with “aged” Aβ_1-42_ peptide conjugated with FITC (BioSource Life Technologies™) with antiretroviral drugs both alone (10 µM) and in combination (10 µM). The total cellular protein of all groups was quantified and adjusted using the Bio-Rad protein assay. Extracellular and cell associated FITC-tagged Aβ was quantified using an SPECTRAmax GEMINI microplate fluorometer (Molecular Devices Corp.) with an emission wavelength of 538 nm and an excitation wavelength of 485 nm. Microglial cells were rinsed 3 times in Aβ-free complete medium, and the media was exchanged with fresh Aβ-free complete medium for 10 min both to allow for removal of non-incorporated Aβ and to promote concentration of the Aβ into phagosomes. The relative mean fluorescence values for each sample at 37°C and 4°C at the indicated time points were determined by fluorometric analysis. Relative mean values were calculated as: (mean fluorescence value for each sample at 37°C - mean fluorescence value for each sample at 4°C). In this manner, both extracellular and cell associated FITC-labeled Aβ were quantified.

### Statistical analysis

All data were normally distributed; therefore, in instances of single mean comparisons, Levene's test for equality of variances followed by *t*-test for independent samples was used to assess significance. In instances of multiple mean comparisons, analysis of variance (ANOVA) was used, followed by *post-hoc* comparison using Bonferonni's method/correction. Alpha levels were set at 0.05 for all analyses. The statistical package for the social sciences release 10.0.5 (SPSS Inc., Chicago, IL, USA) was used for all data analysis.

## Results

Epidemiological reports indicate that HAND persists in patients even with good viremic control who take EFV [3]. Previous studies have shown that cART imparts mitochondrial toxicity in the form of elevate ROS [23], [24]. A high ROS microenvironment has been shown to promote the activity of BACE-1, a key enzyme the generation of Aβ in the brain [52]. Brain oligomeric [53] and Aβ_1-40,42_ [54] have been correlated with cognitive impairment. Since the EFV containing regimen may promote mitochondrial dysfunction [23], [24], [27], [34], [55] which could result in increased BACE-1 activity, we investigated the effect of a commonly used EFV containing cART regimen [29]–[31] for its ability to upregulate Aβ production *via* activation of BACE-1 and amyloidogenic APP processing and also for its ability to reduce microglial phagocytosis of Aβ_1-40,42_.

### BACE-1 is involved in Aβ generation promoted by the EFV containing cART regimen in cultured SweAPP N2a cells (Fig. 1)

**Figure 1:**
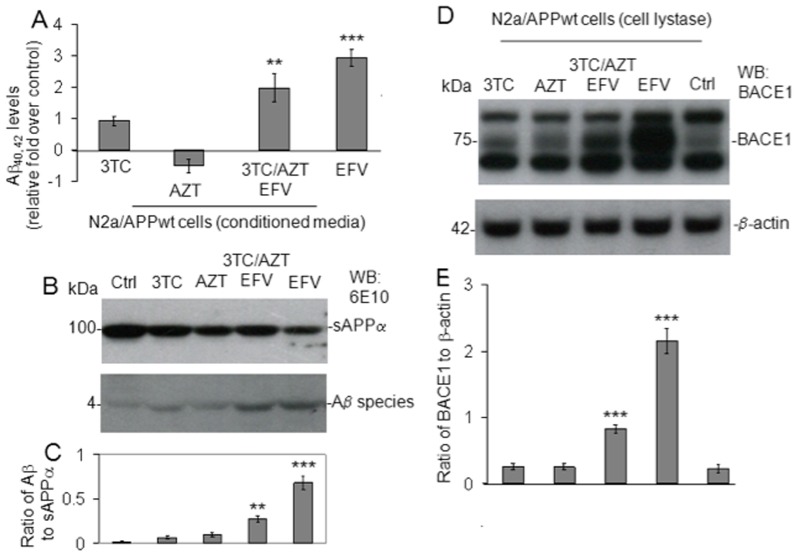
EFV or EFV/3TC/AZT treatment promotes Aβ generation in cultured neuronal cells *via* BACE-1 activation *in vitro.* Aβ species were analyzed in cell lysates from SweAPP N2a cells (**A**) by ELISA. Data are represented as the mean ± of a percentage of Aβ peptides secreted 24 h after 3TC, AZT, EFV, or 3TC/EFV/AZT administration, relative fold over control (PBS treated). Significant increases in Aβ were observed in EFV or EFV/3TC/AZT treated cells were observed compared to control (***P<0.001 and **P<0.05 respectively by ANOVA). (**B**) Western blot (6E10 antibody) of conditioned media shows increased oligomeric Aβ species vs. s-APP-α (control) in the EFV or EFV/3TC/AZT treated cells (***P<0.001 and **P<0.05 respectively). (**D**) BACE-1 expression in cultured media revealed significant differences between EFV or EFV/3TC/AZT treated cells compared to untreated control (***P<0.001). β-actin is used for the internal loading control. Results are representative of three independent experiments.

Using similar conditions as in our prior investigations [44], SweAPP N2a cells were treated with the EFV containing regimen: 3TC, AZT, EFV or each drug singly at 10 µM in addition to PBS control for 18 hours. Aβ_40_ and Aβ_42_ peptides were then measured in conditioned media from these cells by ELISA (Fig. 1A–C) while BACE-1 expression was measured in cell lysates by Western Blot analysis (Fig. 1D–E). The EFV containing regimen increased Aβ_40_ and Aβ_42_ production in SweAPP N2a cells significantly (**P<0.05). Importantly, we found that EFV alone was more potent than the EFV containing regimen in terms of significantly increasing Aβ_40_ and Aβ_42_ production by these cells (***P<0.001). Additionally EFV or the EFV containing regimen increased BACE-1 expression in SweAPP N2a cells significantly (***P<0.001). These data would suggest that 3TC and/or AZT somehow reduces the toxicity of EFV in terms of promoting amyloidogenic APP processing and that EFV is the primary agent promoting Aβ production in SweAPP N2a cells. There is some evidence to indicate that AZT may indeed have a neuroprotective effect [56]–[58] which could explain why the EFV containing regimen is less potent in its amyloid producing effects compared to EFV alone.

### Cerebral amyloidosis in Tg2576 mice is increased by EFV or the EFV containing cART regimen (Fig 2)

Brain Aβ deposition is a pathognomonic feature of AD [59], and oligomeric Aβ species are thought to be a driving force in AD-type neurodegeneration [60]–[63]. They may also play a role in HAND development [37]–[42] The transgenic Tg2576 mouse [64] is a widely used model of cerebral amyloidosis, and we purchased them from Taconic (Germantown, NY) at 8 months of age. They were evaluated for changes in cerebral Aβ after 10 days treatment with each antiretroviral singly or combined as well as vehicle control. Data are represented as mean ± SD with *n* = 5 females per group at 8 months of age. Western blot analysis of brain homogenates revealed significantly increased Aβ species in both the EFV and EFV containing regimen groups (**p<0.01 and 0.05 respectively); again suggesting that EFV accelerates cerebral amyloidosis as opposed to having a cumulative effect with 3TC and AZT. Indeed AZT is most likely behind the reduced potency of the EFV containing regimen compared to EFV alone in terms of Aβ pathology in light of reports that it may be neuroprotective [56]–[58]. Additionally EFV or the EFV containing regimen increased BACE-1 expression in SweAPP N2a cells significantly (***P<0.001).

**Figure 2:**
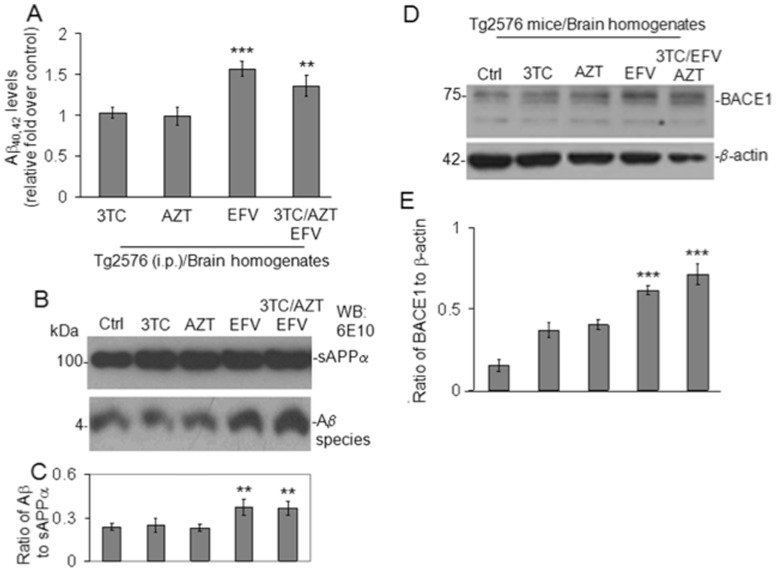
EFV/3TC/AZT increases soluble Aβ levels in Tg2576 mice *via* BACE-1 activation *in vivo.* (**A**) Aβ_40, 42_ peptides were analyzed in brain homogenates from 8 month old Tg2576 mice by ELISA (n = 5 mice for each group). One-way ANOVA followed by *post hoc* comparison revealed significant differences between control (Tg2576mice treated with PBS) and EFV or EFV/3TC/AZT -treated Tg2576 mice (*P<0.001* and *0.05* respectively with n = 5 mice/group). (**B**) Western Blot of brain homogenates using anti-Aβ_1-17_ antibody (6E10) shows total APP and a bands corresponding to soluble Aβ oligomer species. β-actin was an internal control. A *t-test* revealed significant differences in soluble Aβ species between EFV-treated compared to 3TC/AZT/EFV, 3TC or AZT treated Tg2576 mice (*P*<0.01) (**C**) BACE-1 expression in brain homogenate of Tg2576 mice significantly was increased in EFV or EFV/3TC/AZT -treated Tg2576 mice (*P*<0.001).

### EFV promotes mitochondrial stress in SweAPP N2a cells (Fig. 3)

To determine if EFV or the EFV containing cART regimen could promote mitochondrial stress in an amyloid producing model, SweAPP N2a cells were treated with EFV, 3TC, AZT, or all three antiretrovirals combined in addition to vehicle control (PBS) for 48 hours. We performed three separate assays to determine general mitochondrial function. These included analyses of cellular ATP production, MMP, and ROS production. EFV or the EFV containing regimen were most potent in reducing mitochondrial function. Mitochondria produce approximately 90% of the total cellular ATP in neurons [65]. We therefore first examined ATP levels in SweAPP N2a cells as a measure of mitochondrial function. Cells treated with EFV or the EFV-containing regimen had greatly decreased ATP levels (***P<0.001) although the EFV containing regimen had slightly less ATP depletion than EFV alone. Mitochondria from SweAPP N2a cells treated with EFV or the EFV containing regimen showed significantly reduced maximal respiratory rates compared to 3TC or AZT treated SweAPP N2a cells; mirroring the results with the ATP analysis. The MMP is an indicator of electron transport chain function [65].

**Figure 3:**
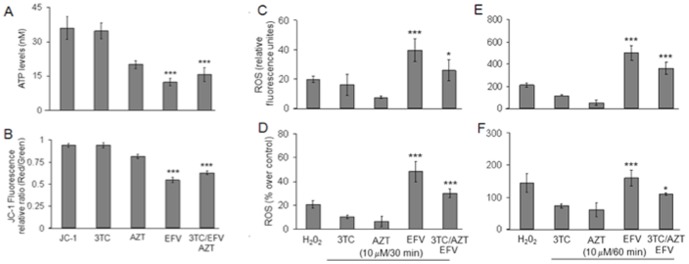
cART treatment of SweAPP N2a cells promotes mitochondrial dysfunction. (*A) ATP levels are reduced in EFV or EFV/3TC/AZT treated SweAPP N2a neuron cells:* SweAPP N2a cells were grown with 10 µM of each medication or all three medications combined for 48 h. We found a significant decrease in ATP levels in cells treated with EFV or 3TC/AZT/EFV (***P<0.001). *(B)MMP is reduced in EFV or EFV/3TC/*AZT *SweAPP N2a cells:* In accord with reduced ATP levels we found a similar reduction in MMP in the EFV or EFV/3TC/AZT treated groups (***P<*0.001*) *(C*–*F) ROS levels are increased in EFV or EFV/3TC/AZT treated SweAPP N2a cells:* EFV-treated primary neuron cells have significantly higher ROS contents (^**^ *P*<0.001) after incubation for 60 min than untreated primary neuron. (C–E)The average relative fluorescence units of DCFDA in neurons from each treatment group as indicated by the mean ± standard deviations (D, F) The ROS content in the antiretroviral treatment is expressed as % RFU ± standard deviations for each group compared to untreated control primary neuron cells (100%). (*P<0.05, *** P<*0.001*).

Mitochondria are the main source of cellular ROS in the brain, thus the rate of ROS reflects the efficiency of mitochondrial function as well [65] (Fig. 3C–F). EFV or the EFV containing regimen caused a large increase in ROS production (*P*<0.001 and *P<*0.05 respectively). AZT and 3TC did not cause a significant rise thus explaining the reduced potency in terms of promoting ROS production of the three drug combination versus EFV alone.

From the three cell-based assays that were utilized to monitor different parameters of mitochondrial function, EFV was identified as the most deleterious compound in our screen of this commonly used cART regimen [29]–[31]. From all three assays we see that AZT and 3TC reduce this effect promoted by EFV.

### Microglial phagocytosis of Aβ_1-42_ peptides is opposed by EFV (Fig. 4)

Amyloid load in the brain is affected not only by production, but also by its clearance from the brain *via* microglia mediated mechanisms [66]. To determine whether the EFV containing regimen could affect microglial clearance of Aβ and further promote amyloidosis, we performed a phagocytosis assay with primary mouse microglia in the presence of EFV, 3TC, AZT or all three antiretrovirals combined in addition to PBS control. Following detection of FITC-tagged Aβ_1-42_ in extracellular and cell associated fractions, we again found that EFV or the EFV containing regimen inhibited microglial phagocytosis/clearance. These two treatments significantly inhibited microglial phagocytosis of Aβ_1-42_ peptides as determined by high levels of peptide remaining in the cultured media (extracellular) (*p<0.001* and *p<0.05 respectively*). In addition, EFV or the EFV containing regimen tested also significantly reduced levels of phagosomal (cell associated) Aβ_1-42_ (*p<0.001* and *p<0.05 respectively*). Also, when comparing cell associated Aβ_1-42_ levels of the EFV compared to the three drug combination to levels of these compound alone, the differences suggest the major reduction in phagocytosis is imparted by EFV and the addition of the other two antiretrovirals of the regimen are not additive in nature. Importantly, when comparing the levels of extracellular Aβ_1-42_ to that of cell associated we can see that the phagocytosis/clearance profiles are relatively congruent for each treatment condition. That is to say, when a given treatment maintains high levels of extracellular Aβ_1-42_, the corresponding cell associated levels are relatively low. Not only does this apparent relationship between extracellular and cell associated Aβ_1-42_ levels confirm the accuracy of the assay, but also furthers the overall significance of the inhibition of microglial phagocytosis by the antiretrovirals [44].

## Discussion

**Figure 4:**
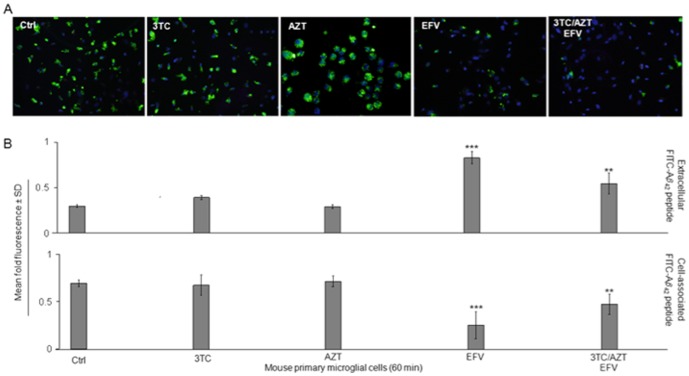
EFV/3TC/AZT inhibits microglial phagocytosis of Aβ_1-42_ peptide. (A) Primary microglia (1×10^5^ cells/well in 24-well tissue culture plates) were treated with aged FITC tagged Aβ_1-42_ (50 nM) in complete medium for 60 min with antiretroviral medications (10uM) combined or singly as indicated, or PBS (control). As a control for nonspecifically incorporated Aβ, microglial cells were incubated at 4°C with the same treatment followed by DAPI staining. EFV or 3TC/AZT/EFV inhibited microglia-colocalization by fluorescence microscopy. Green indicates Aβ_1-42_ positive; blue indicates microglia nuclei. Addition of heat inactivated HIV-1 Tat yielded similar results as vehicle control (data not shown) (B) Cell supernatants and lysates were analyzed for extracellular (top) and cell associated (bottom) FITC-Aβ using a fluorimeter. Data are represented as the relative fold of mean fluorescence change (mean ± SD), calculated as the mean fluorescence for each sample at 37°C divided by mean fluorescence at 4°C (*n* = 6 for each condition presented). One-way ANOVA followed by *post-hoc* comparison showed a significant difference between EFV (***P<0.001) or EFV/3TC/AZT (**P<0.05) but not 3TC or AZT compared to control.

**Figure 5:**
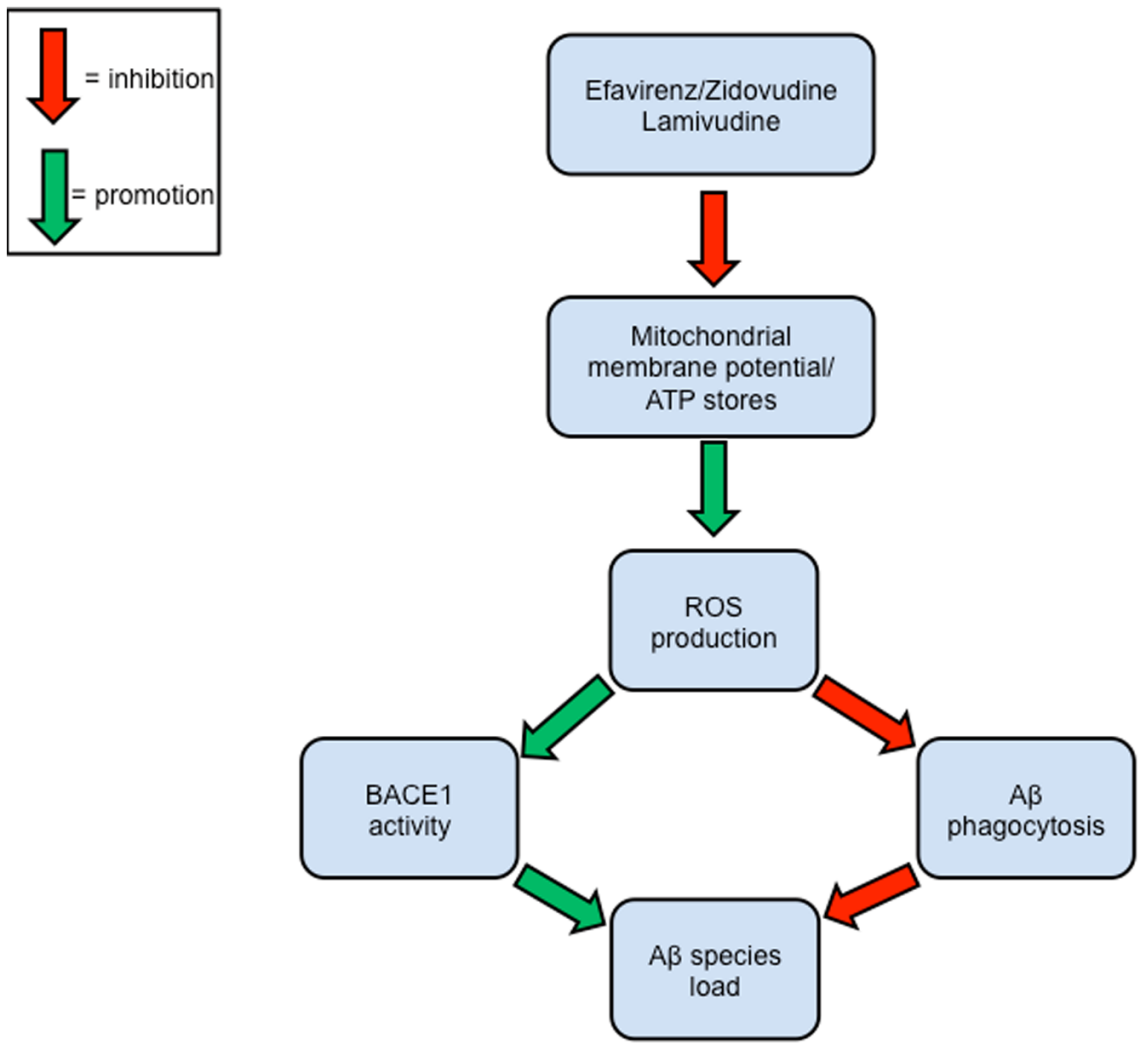
Proposed mechanism of EFV neurotoxicity. Our present work suggests that EFV promotes an increase in Aβ *in vitro* and *in vivo* on both the production and clearance fronts *via* its inhibition of neurnoal MMP resulting in reduced ATP stores and thus a high ROS environment in the CNS. Previous studies indicate such high ROS microenvironments in the CNS promote BACE-1 APP processing and also inhibit microglial Aβ clearance functions. These events in turn all promote production of Aβ species. (*Note: Red arrows = inhibition, Green arrows = promotion).

Here, we elucidate a potential mechanism whereby EFV may have neurotoxic effects *via* promotion of brain Aβ. As shown in Figure 5, our present study has led to the proposed mechanism of neurotoxicity in which EFV promotes an increase in Aβ *in vitro* and *in vivo* on both the production and clearance fronts *via* its inhibition of proper MMP resulting in reduced ATP stores and thus a high ROS environment in the CNS. It is proposed that EFV induced high ROS microenvironments (Fig. 3) in the CNS promote BACE-1 APP processing ([18]; Fig. 1) and also inhibits microglial phagocytic functions (Fig. 4; [67]). These events in turn all promote production of Aβ species.

EFV has been associated with serious adverse reactions, most of which can in part be attributed directly or indirectly to dysfunction of mitochondria [22]–[25], [33]. We found that EFV, or the EFV containing regimen consistently and significantly promoted mitochondrial oxidative stress in the form of reduced cellular ATP stores (Fig. 3A) and MMP (Fig. 3B), as well as increased release of ROS (Fig. 3C–F). These observations suggest the mitochondrial stress imparted by this cART regimen is largely dependent upon EFV and that 3TC and/or AZT may have some protective effect. Indeed there is some evidence that the latter antiretroviral may help to preserve cognitive function [56]–[58]

Reactive microgliosis can be associated with the formation of microglial phenotypes that are unfavorable to phagocytic activities [68]. ROS are an important signal for cellular activation and proliferation. Over the long term lead to microglial dysfunction, rendering the phagocytes unable to perform their vital clearance functions [68]. This may underlie the reduced microglial phagocytosis of Aβ observed in microglia treated with EFV or the EFV containing regimen (Fig. 4).

Several lines of epidemiological evidence signal a role for Aβ in HAND development while some studies have not yet fully implicated over production of the protein as a contributor to HAND. It is known that pathological similarities exist between HAND and AD [37]–[42]. The latter is more so characterized by extracellular deposits of Aβ_1_-_42_ in the form of plaques and aggregations of microtubule-associated tau yielding neurofibrillary tangles (NFT). In contrast, with HIV infection, the plaques are more diffuse [38] rather than neuritic [41].

Cerebrospinal fluid (CSF) biomarkers can mirror pathogenic cerebral amyloid deposition. Decreased CSF Aβ_1-42_ and increased CSF tau can differentiate symptomatic AD participants and cognitively normal individuals at high risk for symptomatic AD from cognitively normal individuals at low risk for symptomatic AD [69], [70]. In that regard, at least some HAND patients have CSF Aβ_1-42_ values comparable to symptomatic AD individuals, that is, reduced [34], [41]. This is salient because reductions in CSF Aβ_42_ have been found in almost all individuals with increased fibrillar amyloid deposition within the brain as assessed with positron emission tomography (PET) amyloid binding of N-methyl-[11C]2-(4-methylaminophenyl)-6-hydroxybenzothiazole (^11^C-PiB) [71]–[73]. Likewise, AIDS dementia complex (ADC) patients had significantly decreased CSF Aβ_1_-_42_ and increased total and phospho (t-tau and p-tau respectively) concentrations similar to AD [38]. Achim and colleague's (2009) reported increased Aβ by both autopsy examination and PET imaging of HIV patients. Specifically, cases with HIV encephalitis (HIV-E) were about twice as likely to have amyloid detected (72%) than HIV+ patients without HIV-E (38%; [37]). In the same year Clifford and colleagues reported Aβ_1_-_42_ measurements in CSF of cognitively impaired patients with HIV were similarly reduced as in in patients with mild dementia of the Alzheimer type (DAT). Normal or slightly depressed CSF tau and p-tau measurements distinguished these patients with HAND from patients with DAT [42].

Further analysis as to why low CSF Aβ_1-42_ in patients with HAND is needed. However, there are several reasons which may explain altered Aβ metabolism in HIV disease [42] in addition to the data presented in this report. First, HIV-1 transactivator of transcription (Tat) protein may compete with APP and/or apolipoprotein E (an Aβ chaperone) for binding to the low density lipoprotein receptor related protein (LRP), thus inhibiting LRP mediated clearance of Aβfrom brain interstitial fluid to periphery [74]. Second, APP cleavage products (sAPPαand sAPPβ) have been reported to be reduced in the CSF of patients with HAND compared to those with DAT or HIV-negative controls, with sAPPα (a neurotrophic protein) showing a slight decline in the asymptomatic HIV state [75].

In 2010 Ances and colleagues reported cognitively unimpaired HIV+ participants, even with low CSF Aβ_1_-_42_ (<500 pg/mL), did not have (11)C-PiB parameters suggesting brain fibrillar amyloid deposition. This dissimilarity between cognitively unimpaired HIV+ and preclinical AD may reflect differences in Aβ_1_-_42_ production and/or formation of diffuse plaques [76]. This same group, in 2012, reported symptomatic AD patients were significantly older, had significantly lower CSF Aβ_1-42_, and had significantly higher CSF tau levels than other groups. Regardless of degree of impairment, HIV patients did not have increased ^11^C-PiB [77]. Possible reasons for the absence of ^11^C-PiB in HIV patients are: 1) decreased Aβ_1_-_42_ production secondary to decreased synaptic activity, 2) increased intraneuronal Aβ_1-42_ deposition that is undetectable by ^11^C-PiB [37]; and/or 3) increased Aβ_1-42_ brain deposition but in a more diffuse, non-fibrillar form that is undetectable by ^11^C-PiB [36], [39]. Future longitudinal examinations within older HIV+ participants are required to determine if diffuse or oligomeric forms could with time subsequently become fibrillar (^11^C-PiB positive) deposits [38], [42]. Our findings reinforce the importance of understanding the effects of cART on amyloid metabolism since EFV could contribute to the neurological complications that are associated with HIV infection seen clinically [3]–[9].

The current research has several strengths and weaknesses. Regarding the former, we observed consistent findings in both *in vitro* and *in vivo* model systems in that EFV or the EFV containing regimen caused increase amyloidogenic APP processing as a function of increased BACE-1 expression and decreased microglial clearance of Aβ. Additionally, we find the level of mitochondrial dysfunction imparted by each antiretroviral medication correlates consistently with the increased level of BACE-1 expression and Aβ production, and the decreased microglial phagocytosis of Aβ peptide. Second our results coincide with other reports indicating the mitochondrial toxicity of antiretrovirals [23]–[27], [32]–[34], and reports that increased ROS can result in increased BACE-1 activity [22].

This report has limitations as well. First, it describes a mechanism for a subset of HAND cases since not all HIV infected individuals are taking EFV or an EFV containing regimen. It should be noted that in the present study, we did not investigate the plasma or CSF concentrations of antiretrovirals or their metabolites. However, all three drugs seem to have good CNS penetration [22], [78], [79], which could support the the neurologic symptoms [3], [5], [8] noted by others.

In sum, our present work suggests that EFV promotes an increase in A*β* on both the production and clearance fronts through oxidative stress. We hypothesize that a disrupted MMP with resultant lowered neuronal ATP stores promotes a high level of ROS. In turn, this can both promote BACE-1 activity and impair microglial clearance mechanisms. If this mouse model translates to the clinical syndrome, then a pharmacotherapeutic strategy aimed at opposing the EFV-mediated reduced microglial A*β* clearance and/or EFV-mediated neuronal A*β* over production *via* BACE-1 should be beneficial to prevent or treat HAND.
